# Long noncoding RNA DLEU2 predicts a poor prognosis and enhances malignant properties in laryngeal squamous cell carcinoma through the miR-30c-5p/PIK3CD/Akt axis

**DOI:** 10.1038/s41419-020-2581-2

**Published:** 2020-06-18

**Authors:** Xiaoming Li, Fenglei Xu, Qiu Meng, Ningyue Gong, Zhenxiao Teng, Runtong Xu, Miaoqing Zhao, Ming Xia

**Affiliations:** 1 Department of Otolaryngology, Shandong Provincial Hospital Affiliated to Shandong First Medical University, No. 324 Jingwuweiqi Road, 250021 Jinan, Shandong Province China; 2 Department of Pathology, Shandong Provincial Hospital Affiliated to Shandong First Medical University, No. 324 Jingwuweiqi Road, 250021 Jinan, Shandong Province China

**Keywords:** Oncogenes, Long non-coding RNAs

## Abstract

Long noncoding RNAs (lncRNAs) have been identified as potential prognostic tools and therapeutic biomarkers for a variety of human cancers. However, the functional roles and underlying mechanisms of key lncRNAs affecting laryngeal squamous cell carcinomas (LSCCs) are largely unknown. Here, we adopted a novel subpathway strategy based on the lncRNA-mRNA profiles from the Cancer Genome Atlas (TCGA) database and identified the lncRNA deleted in lymphocytic leukemia 2 (DLEU2) as an oncogene in the pathogenesis of LSCCs. We found that DLEU2 was significantly upregulated and predicted poor clinical outcomes in LSCC patients. In addition, ectopic overexpression of DLEU2 promoted the proliferation and migration of LSCC cells both in vivo and in vitro. Mechanistically, DLEU2 served as a competing endogenous RNA to regulate PIK3CD expression by sponging miR-30c-5p and subsequently activated the Akt signaling pathway. As a target gene of DLEU2, PIK3CD was also upregulated and could predict a poor prognosis in LSCC patients. In conclusion, we found that the novel LSCC-related gene DLEU2 enhances the malignant properties of LSCCs via the miR-30c-5p/PIK3CD/Akt axis. DLEU2 and its targeted miR-30c-5p/PIK3CD/Akt axis may represent valuable prognostic biomarkers and therapeutic targets for LSCCs.

## Introduction

Head and neck squamous cell carcinoma (HNSCC) is the fifth deadliest cancer, with about 500,000 new patients diagnosed each year worldwide^1^. The HNSCCs include malignancies within the oral cavity, pharynx, larynx, paranasal sinuses, nasal cavity, and salivary glands. The incidence and mortality of laryngeal squamous cell carcinoma (LSCC), one type of HNSCC, have increased each year^2^. Despite aggressive treatment with surgery, radiation, and chemotherapy, the prognosis remains dismal when a patient is diagnosed with an advanced-stage LSCC^3^. Recent high-throughput sequencing of tumors by the Cancer Genome Atlas (TCGA) Research Network has demonstrated that numerous genomic alterations are significantly correlated with the recurrence and poor prognosis of LSCCs^4^. Although the pathogenesis of LSCCs is extremely complex, tools such as molecular profiling may help us to understand the underlying etiology^4,5^. Therefore, a deeper understanding of the genetic and epigenetic molecular mechanisms of LSCCs is imperative for diagnosis and treatment of this terrible disease.

Long noncoding RNAs (lncRNAs) are transcripts of longer than 200 nucleotides without a protein-coding ability^6^. According to the competitive endogenous RNA (ceRNA) hypothesis, lncRNAs competitively bind to microRNA (miRNA) sites through their miRNA response elements and thus regulate the mRNA expression levels^7^. It is well known that lncRNAs play a critical role in a wide range of biological processes by affecting gene transcription, targeting RNA polymerase II, and regulating splicing^8,9^. Furthermore, accumulating evidence indicates that some lncRNAs are involved in regulating tumor malignant properties via modulating cancer-related signaling pathways^10,11^. Thus, identifying pathways that are competitively regulated by lncRNAs should help to elucidate the pathogenesis of HNSCCs^12,13^. Indeed, numerous studies have emphasized the role of lncRNAs in the pathogenesis of LSCC, and several LSCC-related lncRNAs have recently been identified^14–17^. For example, HOX transcript antisense intergenic RNA (HOTAIR) is related to the promotion of PTEN methylation in LSCCs, and increased expression of HOTAIR is an unfavorable prognostic factor of LSCCs^15^. Similarly, RGMB antisense RNA 1 (RGMB-AS1) is upregulated in LSCC specimens and is associated with poor clinical outcomes in LSCC patients^16^. This study found that the lncRNA RGMB-AS1 regulated LSCC cell proliferation and invasion via the miR-22/NLRP3 axis^16^. However, the precise underlying mechanism by which lncRNAs affect the malignant properties of LSCCs remains to be elucidated.

In the present study, we used a subpathway strategy based on the lncRNA-mRNA profiles from the TCGA database to identify lncRNAs that competitively regulate subpathways in HNSCCs^18^. We hypothesized that the lncRNA deleted in lymphocytic leukemia 2 (DLEU2) might play a crucial role in the malignant progression of LSCCs. To verify this hypothesis, we further analyzed the expression levels of DLEU2 in LSCC clinical specimens and investigated the functional roles of DLEU2 in LSCC growth and metastasis both in vitro and in vivo. The mechanisms underlying the effects of DLEU2 on the miR-30c-5p/phosphatidylinositol-4,5-bisphosphate 3-kinase catalytic subunit delta (PIK3CD)/Akt axis are described below in further detail.

## Materials and methods

### Identification of key lncRNA-mRNAs in HNSCCs

We adopted a novel subpathway strategy to identify lncRNAs competitively regulated functions and the key competitive lncRNAs in HNSCCs from the TCGA database (https://www.cancer.gov/tcga). Briefly, the miRNA−mRNA interactions and lncRNA−miRNA intersections were primarily collected from the starBase v2.0 (http://starbase.sysu.edu.cn/), TarBase (http://diana.imis.athena-innovation.gr/DianaTools/index.php?r=tarbase/index), mirTarBase (http://mirtarbase.mbc.nctu.edu.tw/), mir2Disease (http://www.miR2Disease.org) and miRecordsV4.0 (http://c1.accurascience.com/miRecords/), and the lncRNA−miRNA−mRNA network was established through the shared miRNA^19^. Next, we performed the hypergeometric test, Jaccard coefficient standardization and Pearson coefficient to determine the valid interaction relationship. After embedding the lncRNA interactions to pathways according to the Kyoto Encyclopedia of Genes and Genomes (KEGG, https://www.kegg.jp) enrichment analysis, the lncRNA competitively regulated signal pathways (LRSPs) were recognized by the lenient distance and the Wallenius approximation methods^20^. Detailed informations of these processes are described in the Supplementary Materials and Methods.

### Human tissue specimens

The Ethics Committee of the Shandong Provincial Hospital Affiliated to Shandong First Medical University approved the present study. All patients provided their written consent in this study. Detailed informations of the patient samples are described in the Supplementary Materials and Methods. Clinicopathological characteristics of these patients are listed in Table 1.

**Table 1:** Correlation of lncRNA DLEU2 expression with clinical variables in LSCC patients.

Variables	*N*	LncRNA DLEU2 expression		*P* value
		Low	High	
Age (years)				
<60	27	13	14	0.806
≥60	39	20	19	
Gender				
Male	61	30	31	0.648
Female	5	3	2	
Smoking status				
Smokers	43	21	22	0.800
Non-smokers	23	12	11	
Tumor stage				
T1−T2	41	25	16	0.022
T3−T4	25	8	17	
Lymph node metastasis				
Absent	12	10	2	0.010
Present	54	23	31	
TNM stage				
I−II	10	9	1	0.005
III−IV	56	24	32	
Differentiation				
Well to moderate	41	23	18	0.210
Poor	25	10	15	

*TNM* tumor node metastasis.

### Quantitative reverse transcription polymerase chain reaction (qRT-PCR)

Detailed informations of RNA isolation and qRT-PCR were described as reported previously^21^. The relative level of the mRNA was calculated by the 2^−△△CT^ method^22^. The primers are listed in Supplementary Table S1. The results were repeated three times.

### Western blotting

Detailed informations of western blotting are described in the Supplementary Materials and Methods. The results were repeated three times.

### Immunohistochemical (IHC) staining

The IHC staining was performed and evaluated as we previously described^23^. The antibodies for IHC staining are described in the Supplementary Materials and Methods.

### Plasmid construction, cell transfection and cell culture

The pcDNA3.1 plasmid with DLEU2 overexpression (Ov-DLEU2) and pcDNA3.1 empty plasmid (Ov-Ctrl) were purchased from Tolo Biotech (Shanghai, China). Lentiviral plasmid with short-hairpin RNA of DLEU2 (sh-DLEU2), short-hairpin RNA of PIK3CD (sh-PIK3CD) and nontargeting plasmids (sh-Ctrl) were designed and purchased from GenePharma (Shanghai, China). The miR-30c-5p mimics and negative control mimics were purchased from RiboBio Co. Ltd. (Guangzhou, China). Plasmid transfections were performed using Lipofectamine 2000 (Invitrogen, Carlsbad, CA) according to the manufacturer’s instructions. Human LSCC lines Hep2 and AMP-HN-8 were purchased from the Cell Bank of Chinese Academy of Sciences (Shanghai, China) and cultured in Dulbecco’s Modified Eagle’s medium supplemented with 10% fetal bovine serum at 37 °C in a 5% CO_2_ cell culture incubator.

### Cell viability measurement, ethynyl deoxyuridine (EdU) assay and colony formation assay

The cell viability was measured by the Cell Counting Kit 8 (CCK8) in accordance with the manufacturer’s instructions (Apexbio, Houston, USA). The EdU assay was performed with an EdU kit (Roche, Indianapolis, IN, USA) according to the manufacturer’s instructions and analyzed with a flow cytometer by the CellQuest software (BD Biosciences, CA, USA). Detailed informations of the colony formation assay are described in the Supplementary Materials and Methods. These experiments were repeated three times.

### Migration and invasion assays

Detailed informations of the migration and invasion assays are described in the Supplementary Materials and Methods. The results were repeated three times.

### Dual-luciferase reporter gene assay

Detailed informations of the dual-luciferase reporter gene assay are described in the Supplementary Materials and Methods. The results were repeated three times.

### In vivo tumor xenograft and metastatic model

All animal experiments were approved by the Animal Care and Use Committee of Shandong Provincial Hospital Affiliated to Shandong First Medical University. The xenograft models were performed as described previously^23^. Detailed informations of the in vivo lung metastasis assay are described in the Supplementary Materials and Methods.

### Statistical analysis

Statistical analyses were performed by SPSS 13.0 software (SPSS, Chicago, IL, USA). Student’s *t* test, one-way ANOVA, the Mann–Whitney *U* test and the *x*^2^ test were used to analyze differences among different groups. Survival curves were plotted by the Kaplan−Meier method and evaluated by the log-rank test. The Cox proportional hazards regression models were used to determine the independent prognostic factors in LSCC patients. All the results were presented as the mean ± SD and *P* values < 0.05 were considered statistically significant.

## Results

### Identification of key lncRNAs and the corresponding target genes in HNSCCs

**Fig. 1:**
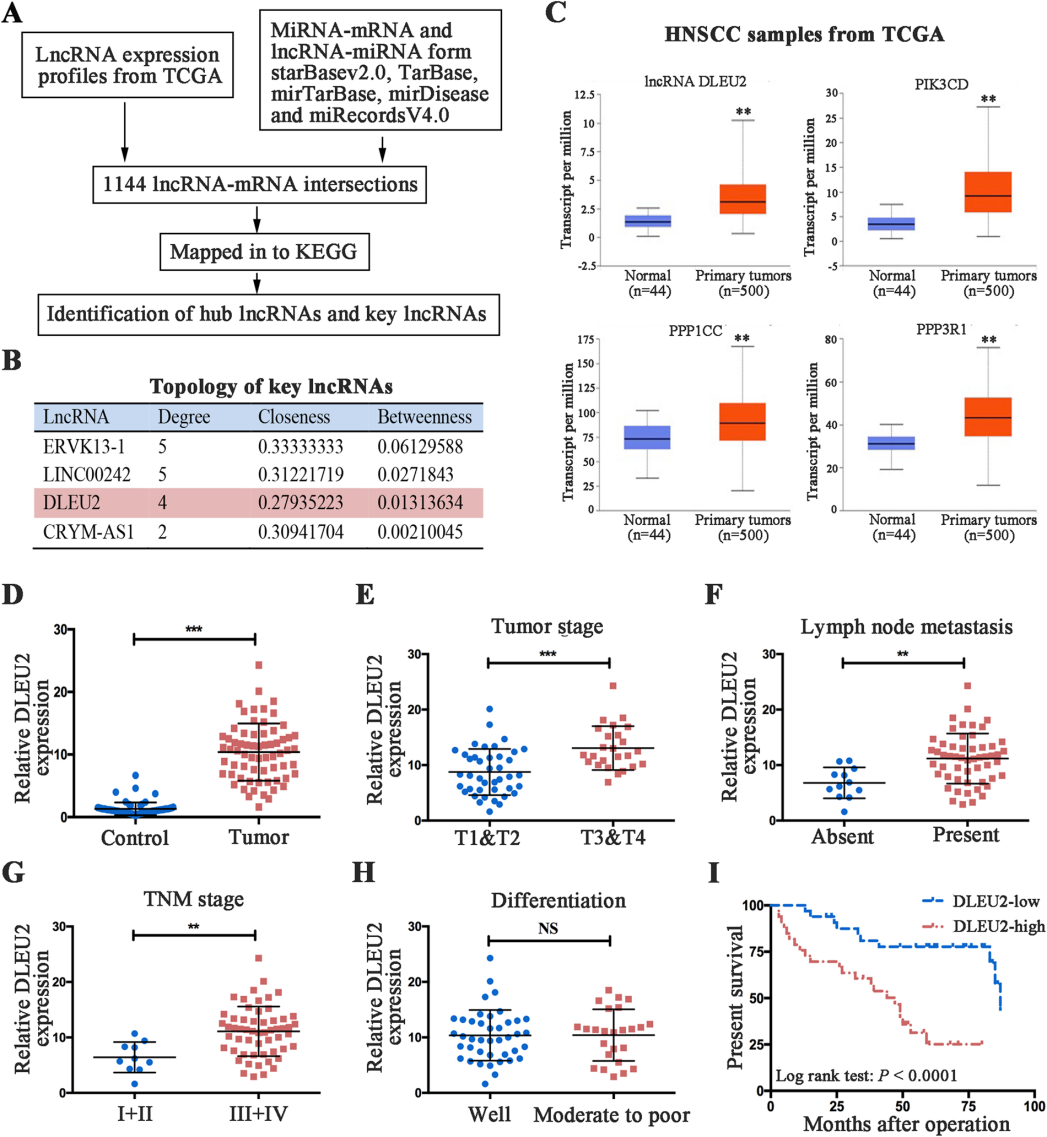
DLEU2 is a novel LSCC-related oncogene and predicts the prognosis in LSCC patients. **a** Schematic depicting key lncRNAs and hub genes in the LRSP subpathways and network. **b** Topology features of the key lncRNAs. **c** DLEU2 and its potential target genes (PIK3CD, PPP1CC, and PPP3R1) were significantly upregulated in the tumor tissues compared with the normal control tissues in HNSCC patients from the TCGA database. **d** The mRNA expression of DLEU2 was evaluated by qRT-PCR analysis in 66 cases of LSCC specimens and the matched adjacent normal tissues. **e–h** Correlations of DLEU2 expression with the clinicopathological characteristics of the tumor stage (**e**), lymph node metastasis (**f**), tumor node metastasis (**g**), and differentiation (**h**) in LSCC specimens. **i** The correlation between survival time and DLEU2 expression was analyzed in LSCC patients. **P < 0.01, ***P < 0.001.

We adopted a novel subpathway strategy through multiple microarrays and diverse bioinformatics platforms to identify key lncRNAs in HNSCCs (Fig. 1a). After matching the lncRNA−mRNA interactions to the KEGG database, the signaling pathways competitively regulated by lncRNA were obtained (Supplementary Fig. S1 and Supplementary Table S2). Next, we built the lncRNA−mRNA interaction networks, and a total of 19 hub lncRNAs were identified (Supplementary Fig. S2). The molecules with roles in the top three LRSP subpathways were identified as: DLEU2, endogenous retrovirus group K13 member 1 (ERVK13-1), long intergenic nonprotein coding RNA 242 (LINC00242), and crystallin mu antisense RNA 1 (CRYM-AS1). The network of key lncRNA–mRNA interactions is shown in Supplementary Fig. S3. The topology of this map indicated that the degree of CRYM-AS1 was less than the average degree (Fig. 1b). After searching for studies in the literature that included descriptions of these key lncRNAs, we found a limited number of articles that investigated LINC00242 and ERVK13-1. Therefore, the subsequent analysis focused on DLEU2. In order to verify our prediction, we investigated the expression signatures of DLEU2 and its potential target genes (PIK3CD, PPP1CC, and PPP3R1) in HNSCC samples from the TCGA database. The results indicated that DLEU2 and its potential targets were significantly upregulated in HNSCCs (Fig. 1c).

### Increased expression of DLEU2 is associated with tumor malignancies and predicts a poor prognosis in LSCC patients

To validate the clinical significance of DLEU2 in HNSCCs, we selected LSCC, one of the most common HNSCCs, for further analysis. We measured the expression levels of DLEU2 in LSCC clinical specimens and the matched adjacent normal tissues. The results indicated that DLEU2 was significantly upregulated in the tumor tissues, compared with the normal tissues (Fig. 1d). To determine the relationship between DLEU2 expression and LSCC malignancies, the patients were stratified according to the relative mRNA expression of DLEU2 as low-DLEU2 expression (below the median value, *n* = 33) or high-DLEU2 expression (above the median value, *n* = 33). As shown in Table 1, statistically significant correlations were found between high levels of DLEU2 expression and an advanced tumor stage (*P* = 0.022, Fig. 1e), lymph node metastasis (*P* = 0.010, Fig. 1f), and an advanced tumor node metastasis (TNM) stage (*P* = 0.005, Fig. 1g). However, no relationship was found between DLEU2 expression and clinical variables such as age, gender, smoking status, and tumor differentiation (Table 1 and Fig. 1h).

To verify the prognostic value of DLEU2 expression, Kaplan–Meier analyses for overall survival were performed for high- vs. low-DLEU2 expression among patients with LSCC. As shown in Fig. 1i, the overall survival rates were significantly better in patients with low-DLEU2 expression than in those with high-DLEU2 expression (log-rank test: *P* < 0.0001). Univariate analysis indicated that the tumor stage (*P* < 0.0001), lymph node metastasis (*P* = 0.028), and TNM stage (*P* = 0.022) were also prognostic factors for overall survival in LSCC patients. However, the clinical variables such as age, gender, smoking status, and tumor differentiation showed no correlation with overall survival among patients with LSCC (Table 2). In addition, multivariate analysis identified tumor stage (*P* < 0.0001) and DLUE2 expression (*P* = 0.017) as independent prognostic factors in LSCCs (Table 2).

**Table 2:** Univariate and multivariate analyses of overall survival in LSCC patients.

Clinical variables	Unadjusted *P* value	Adjusted	
		Hazard ratio (95% CI)	*P* value
Age			
<60 vs, ≥60	0.176	1.941 (0.774–4.869)	0.157
Gender			
Male vs. female	0.315	0.917 (0.112–7.497)	0.936
Smoking status			
Smokers vs. non-smokers	0.505	0.914 (0.404–2.068)	0.828
Tumor stage			
T1−T2 vs. T3−T4	0.000	4.777 (2.040–11.19)	0.000
Lymph node metastasis			
Absent vs. present	0.028	2.029 (0.247–16.64)	0.510
TNM stage			
I−II vs. III−IV	0.022	1.464 (0.085–25.13)	0.793
Differentiation			
Well vs. moderate to poor	0.167	1.147 (0.488–2.696)	0.754
LncRNA DLEU2 expression			
Low vs. high	0.001	2.985 (1.214–7.341)	0.017

*TNM* tumor node metastasis, *CI* confidence interval.

### DLEU2 promotes LSCC cell proliferation, invasion, and migration in vitro

**Fig. 2:**
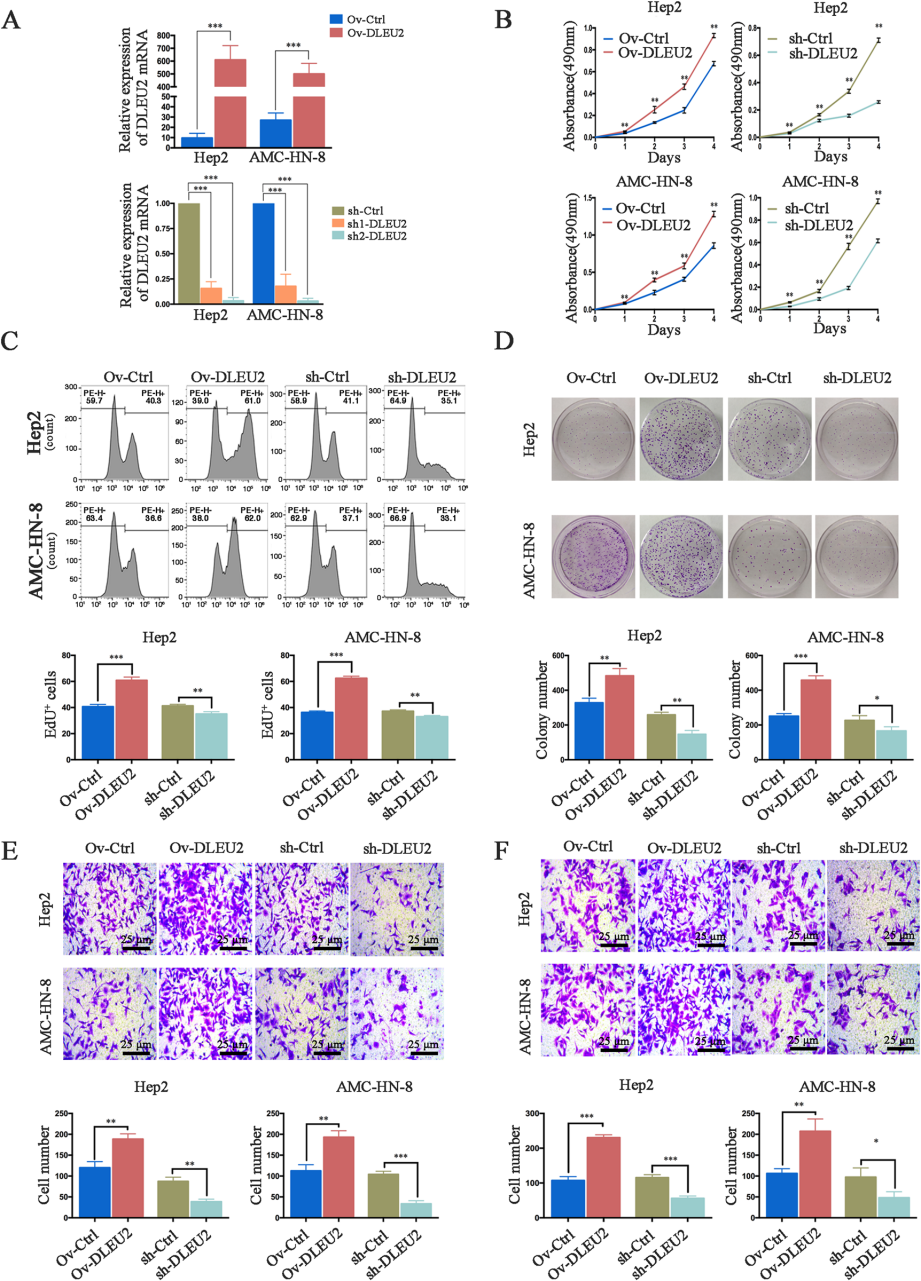
DLEU2 modulates LSCC cell proliferation, invasion, and migration in vitro. **a** The efficiency of transfection was detected by qRT-PCR, as indicated. **b** The CCK-8 assay was performed to evaluate the cell viability at various time points in the indicated cells. **c**, **d** The EdU proliferation assay (**c**) and colony formation assay (**d**) were performed to evaluate the effect of DLEU2 on the proliferation of the indicated cells. **e**, **f** The transwell assay was performed to evaluate the invasion (**e**) and migration (**f**) abilities of the indicated cells (original magnification: ×200; scale bar = 25 µm). The results are presented as the mean ± SD from three independent experiments. *P < 0.05, **P < 0.01, ***P < 0.001.

To illustrate the functional roles of DLEU2 in LSCC cells, we overexpressed or silenced DLEU2 in two LSCC cell lines, Hep2 and AMC-HN-8 (Fig. 2a). We next evaluated the vitality of these two cell lines by performing the CCK-8 assay. The results indicated that DLEU2 overexpression significantly enhanced the vitality of the tumor cells, while DLEU2 knockdown suppressed it (Fig. 2b). In parallel, the EdU proliferation assay and colony-forming assay revealed that DLEU2 overexpression promoted the proliferation of LSCC cells, while DLEU2 knockdown inhibited it (Fig. 2c, d). We also examined the effects of DLEU2 on the invasion and migration of LSCC cells. DLEU2-overexpressing cells appeared to be more invasive than the control cells, and inhibition of DLEU2 appeared to decrease the invasiveness of the LSCC cells (Fig. 2e). Similarly, DLEU2 overexpression enhanced the migration ability of LSCC cells, compared to the control cells. However, DLEU2 knockdown attenuated the migration ability of LSCC cells (Fig. 2f). Taken together, these data indicate that DLEU2 promotes the proliferation, invasion, and migration of LSCC cells.

### PIK3CD is a target gene of DLEU2 and predicts the prognosis in LSCC patients

**Fig. 3:**
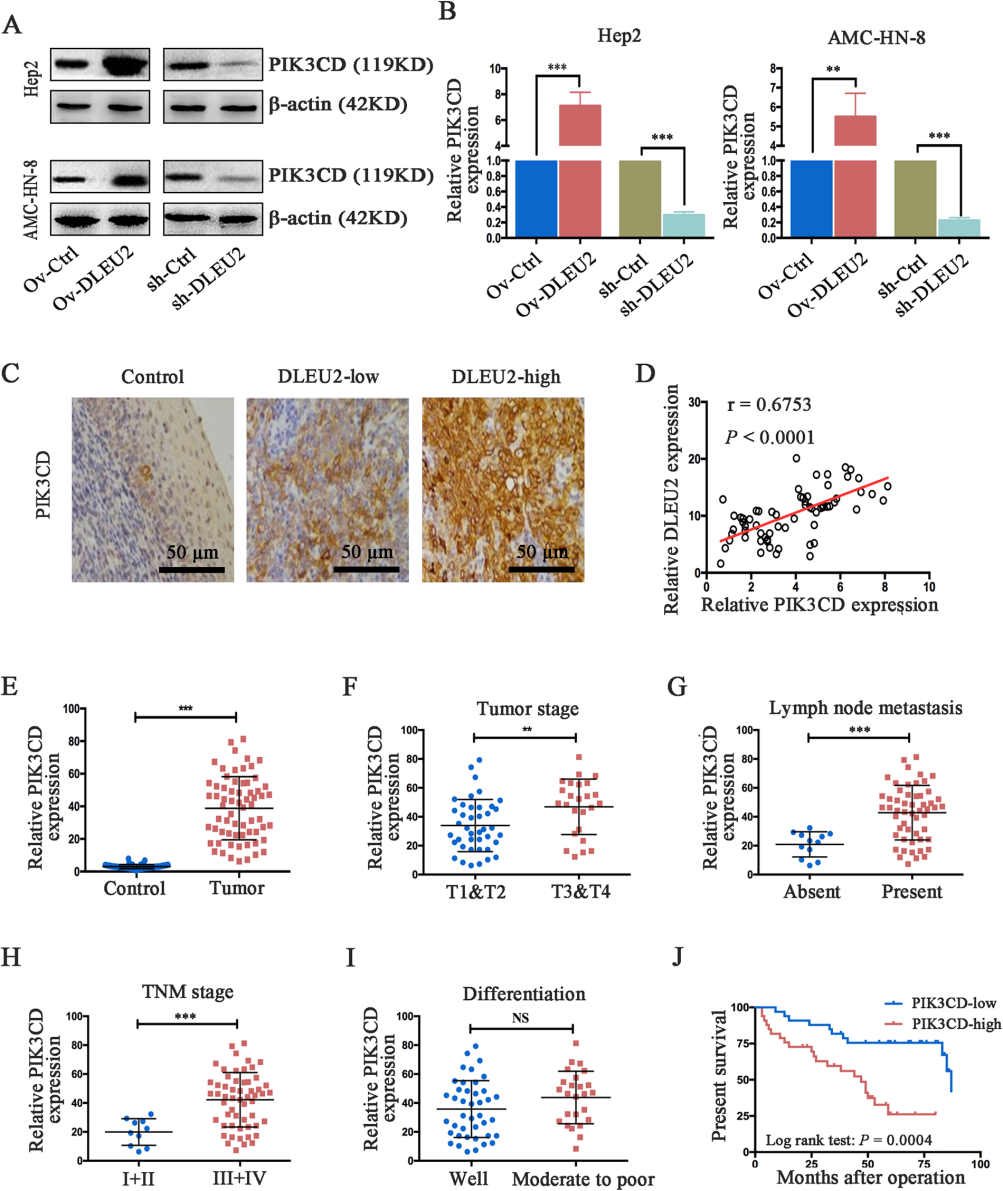
DLEU2 regulates PIK3CD expression, which is correlated with tumor progression and survival time in LSCC patients. **a**, **b** DLEU2 was overexpressed or knocked down in LSCC cells. Western blot (**a**) and qRT-PCR (**b**) analysis were performed to detect PIK3CD expression. β-actin was used as an internal control. **c** Representative immunohistochemical staining of PIK3CD in normal control, low DLEU2 expression, and high DLEU2 expression samples, respectively (original magnification: ×200; scale bar = 50 µm). **d** Spearman’s correlation analysis was performed to evaluate the relationship between the mRNA levels of DLEU2 and PIK3CD in 66 LSCC specimens. **e** The mRNA levels of PIK3CD in 66 LSCC specimens and matched adjacent normal tissues were evaluated by qRT-PCR analysis. **f**–**i** Correlations between PIK3CD expression and the tumor stage (**f**), lymph node metastasis (**g**), tumor node metastasis (**h**), and differentiation (**i**) in LSCC specimens. **j** The correlation between survival time and PIK3CD expression was analyzed in LSCC patients. **P < 0.01, ***P < 0.001.

We next analyzed the interaction network to identify possible targets of DLEU2 (Supplementary Fig. S3) and considered PIK3CD as a potential target of DLEU2 for further investigation. Therefore, we examined the expression levels of PIK3CD in LSCC cells with various levels of DLEU2 expression. Compared to the control cells, the protein and mRNA expression levels of PIK3CD (Fig. 3a, b) were significantly upregulated in cells overexpressing DLEU2 but decreased in DLEU2-silenced cells. Along similar lines, IHC analysis of the LSCC clinical specimens revealed a positive association between DLEU2 and PIK3CD (Fig. 3c). We also measured the mRNA level of PIK3CD in clinical LSCC samples. The results indicated that PIK3CD expression was positively correlated with DLEU2 expression (Spearman’s correlation test, *r* = 0.6753, *P* < 0.0001, Fig. 3d). These results suggest that PIK3CD expression is upregulated by DLEU2 in LSCC cells.

PIK3CD has been reported to be a key regulator of malignant progression in various human cancers^24–29^. Therefore, we further analyzed the correlations of PIK3CD expression with the clinical variables in LSCC patients. We found that PIK3CD expression was significantly upregulated in the LSCC tissues, compared with the adjacent normal tissues (Fig. 3e). Moreover, the LSCC patients with an advanced tumor stage, the presence of lymph node metastasis and a higher TNM stage showed increased PIK3CD expression, compared to the matched group of controls (Fig. 3f–h). However, a significant correlation between PIK3CD expression and differentiation was not found in the present study (Fig. 3i). Furthermore, the Kaplan–Meier analysis indicated that low-PIK3CD expression predicted a better overall survival in patients with LSCC (log-rank test: *P* = 0.0004, Fig. 3j). These results demonstrate that the acceleration of LSCC malignant progression by DLEU2 is at least partly mediated by PIK3CD.

### DLEU2 acts as a molecular sponge for miR-30c-5p to upregulate PIK3CD

**Fig. 4:**
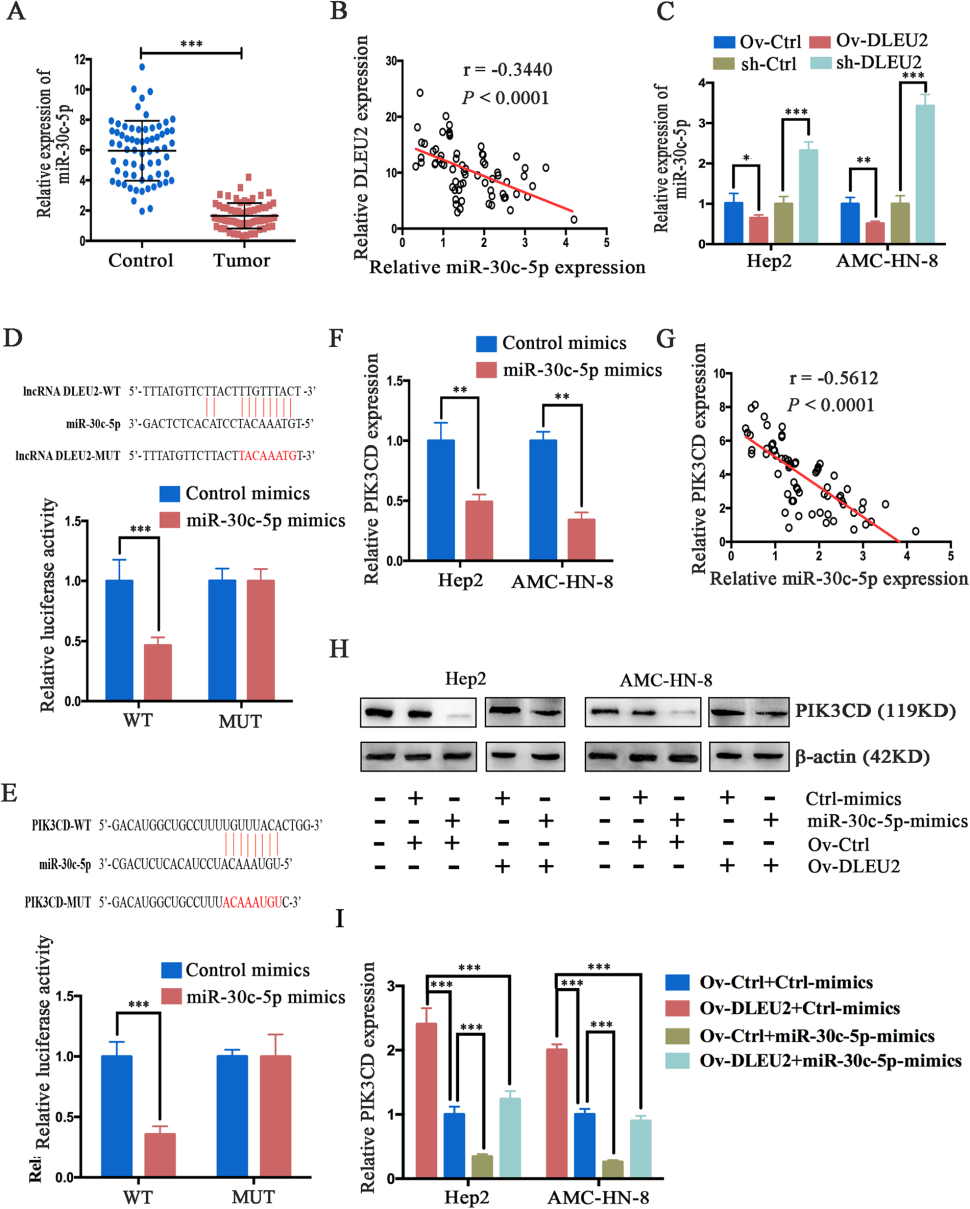
DLEU2 functions as a competing endogenous RNA and regulates PIK3CD expression by sponging miR-30c-5p in LSCC cells. **a** The mRNA expression of miR-30c-5p was measured by qRT-PCR analysis in 66 LSCC specimens and matched adjacent normal tissues. **b** Spearman’s correlation analysis was used to evaluate the relationship between mRNA expression of DLEU2 and miR-30c-5p in 66 LSCC specimens. **c** After DLEU2 was overexpressed or knocked down in LSCC cells, qRT-PCR analysis was performed to measure the levels of miR-30c-5p mRNA. β-actin was used as an internal control. **d**, **e** The putative miR-30c-5p binding sites in the lncRNA DLEU2 (**d**) and the PIK3CD (**e**) sequences (PI3KCD-WT), as predicted by TargetScan, and the mutant sequences designed (lncRNA DLEU2-MUT and PIK3CD-MUT). Luciferase activity was determined by a luciferase reporter assay to confirm the direct correlation among the levels of miR-30c-5p, DLEU2, and PIK3CD. **f** QRT-PCR was performed to measure the PIK3CD expression of the indicated cells. **g** Spearman’s correlation analysis was used to evaluate the relationship between miR-30c-5p and PIK3CD mRNA expression in 66 LSCC specimens. **h**, **i** DLEU2 overexpression increased the protein (**h**) and mRNA (**i**) expression levels of PIK3CD. This effect was reversed by the induction of miR-30c-5p expression in LSCC cells. *P < 0.05, **P < 0.01, ***P < 0.001.

To further investigate the underlying mechanism by which DLEU2 regulates PIK3CD, bioinformatics tools were used to analyze the lncRNA–miRNA–mRNA network. We found that miR-30c-5p appeared to be a potential link between DLEU2 and PIK3CD. We first analyzed miR-30c-5p expression in LSCC clinical specimens by qRT-PCR. The results indicated that miR-30c-5p expression was significantly downregulated in LSCC tissues (Fig. 4a) and negatively correlated with DLEU2 expression (Spearman’s correlation test, *r* = −0.3440, *P* < 0.0001, Fig. 4b). Meanwhile, miR-30c-5p expression was markedly decreased in cells overexpressing DLEU2 but elevated in DLEU2-silenced cells (Fig. 4c). Moreover, TargetScan predictions revealed that both the 3ʹ-untranslated region (UTR) of DLEU2 and the 3ʹ-UTR of PIK3CD harbored a putative miR-30c-5p binding site. The existence of these binding sites was confirmed by the luciferase reporter assay (Fig. 4d, e). Functionally, overexpression of miR-30c-5p inhibited PIK3CD mRNA expression in LSCC cells (Fig. 4f), and miR-30c-5p expression was inversely correlated with PIK3CD expression in LSCC clinical specimens (Spearman’s correlation test, *r* = −0.5612, *P* < 0.0001, Fig. 4g). Additionally, gain-of-function experiments indicated that the upregulation of PIK3CD induced by DLEU2 overexpression was attenuated by miR-30c-5p (Fig. 4h, i). Taken together, all these results indicate that DLEU2 functions as a competing endogenous RNA to regulate PIK3CD expression by sponging miR-30c-5p.

### DLEU2 activates the Akt signaling via PIK3CD, and silencing of PIK3CD attenuates the oncogenic function of DLEU2

**Fig. 5:**
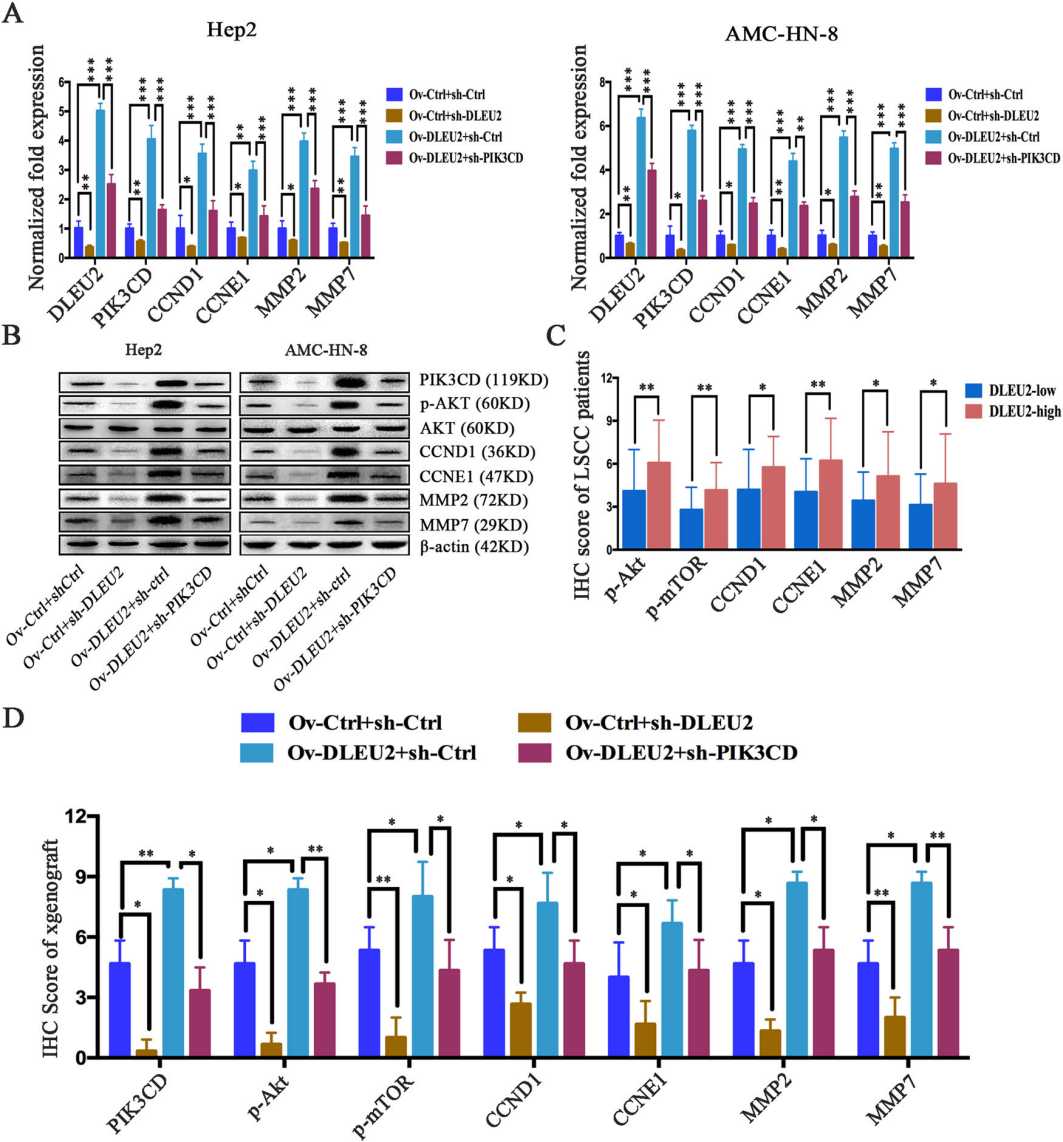
DLEU2 promotes the Akt signaling by upregulating PIK3CD. **a** QRT-PCR analysis of DLEU2, PIK3CD, CCND1, CCNE1, MMP2, and MMP7 in the indicated cells. **b** Western blot analysis of PIK3CD, Akt, p-Akt, CCND1, CCNE1, MMP2, and MMP7 in the indicated cells. **c** Immunohistochemical scores for p-AKT, p-mTOR, CCND1, CCNE1, MMP2, and MMP7 in LSCC specimens, stratified by DLEU2 expression. **d** Immunohistochemical scores for PIK3CD, p-AKT, p-mTOR, CCND1, CCNE1, MMP2, and MMP7 in samples from the xenograft tumors. These results are presented as the mean ± SD from three independent experiments. *P < 0.05, **P < 0.01, ***P < 0.001.

To further investigate the mechanism by which DLEU2 promotes the malignant progression of LSCCs, we estimated the impacts of DLEU2 on PIK3CD and its target genes in LSCC cells. PIK3CD has recently been found to play a critical role in mediating the development of malignant tumors through activation of Akt signaling^24–29^. To address whether Akt signaling targets are regulated by DLEU2, we detected the protein and mRNA levels of Akt and its target genes in LSCC cells expressing various levels of DLEU2 and PIK3CD. Akt targets, including CCND1, CCNE1, MMP2, and MMP9, were significantly enhanced in cells with DLEU2 overexpression, compared to the matched control cells, but remarkably suppressed in cells with DLEU2 knockdown. However, PIK3CD knockdown attenuated this process in LSCC cells (Fig. 5a, b). Consistently, the IHC staining results indicated that the protein expression levels of p-AKT, p-mTOR, CCND1, CCNE1, MMP2, and MMP7 were significantly increased in the high-DLEU2 group of LSCC clinical specimens, compared with the low-DLEU2 group (Fig. 5c). Furthermore, the IHC staining results in xenograft tumors indicated that both PIK3CD and the Akt signaling targets were upregulated by the overexpression of DLEU2 but suppressed by the knockdown of DLEU2. However, PIK3CD knockdown attenuated the upregulation of Akt signaling induced by overexpression of DLEU2 (Fig. 5d). These results suggest that DLEU2 promotes Akt signaling by upregulating PIK3CD.

**Fig. 6:**
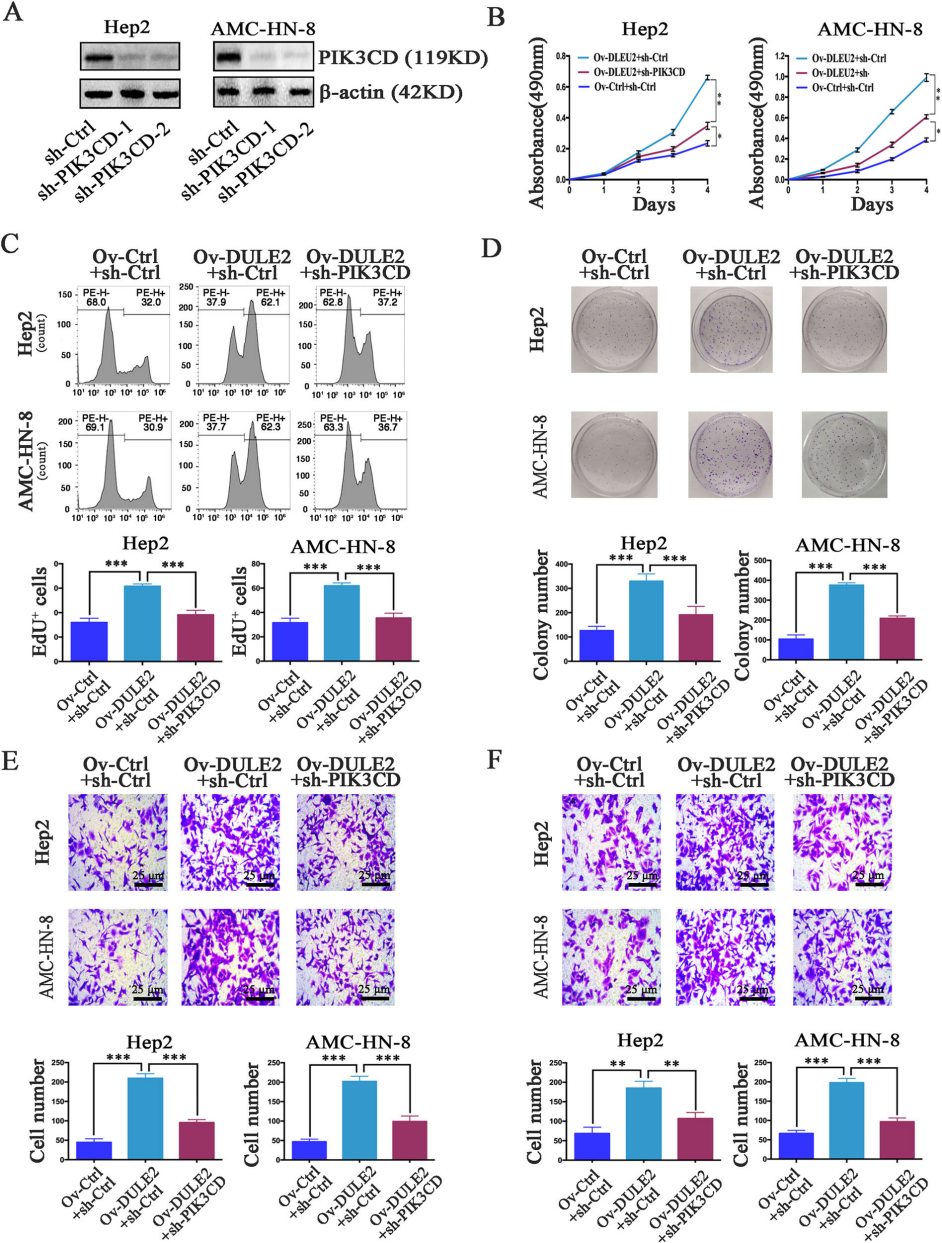
DLEU2 promotes LSCC cell proliferation, invasion, and migration by targeting PIK3CD expression. **a** The expression of PIK3CD was confirmed by western blotting in Hep2 and AMC-HN-8 cells with different expression levels of PIK3CD. **b** The cell vitality was evaluated with the CCK-8 assay at various time points in the indicated cells. **c**, **d** Cellular proliferation was measured with the EdU proliferation assay (**c**) and colony formation assay (**d**) in the indicated cells. **e**, **f** The transwell assay was performed to measure the invasion (**e**) and migration (**f**) abilities of the indicated cells (original magnification: ×200; scale bar = 25 µm). The results are presented as the mean ± SD from three independent experiments. **P < 0.01, ***P < 0.001.

Based on the above observations, we next sought to investigate whether PIK3CD was responsible for the enhanced proliferation, invasion, and migration induced by DLEU2. We silenced PIK3CD in LSCC cells (Fig. 6a) and found that inhibition of PIK3CD could abolish the role of DLEU2 in promoting cell vitality, proliferation, and colony formation (Fig. 6b–d). The increases in cell invasion and migration induced by DLEU2 overexpression were also reversed by PIK3CD knockdown (Fig. 6e, f). These data indicate that PIK3CD knockdown in LSCC cells can inhibit the tumorigenic function of DLEU2.

### Overexpression of DLEU2 promotes tumor growth and metastasis in vivo

**Fig. 7:**
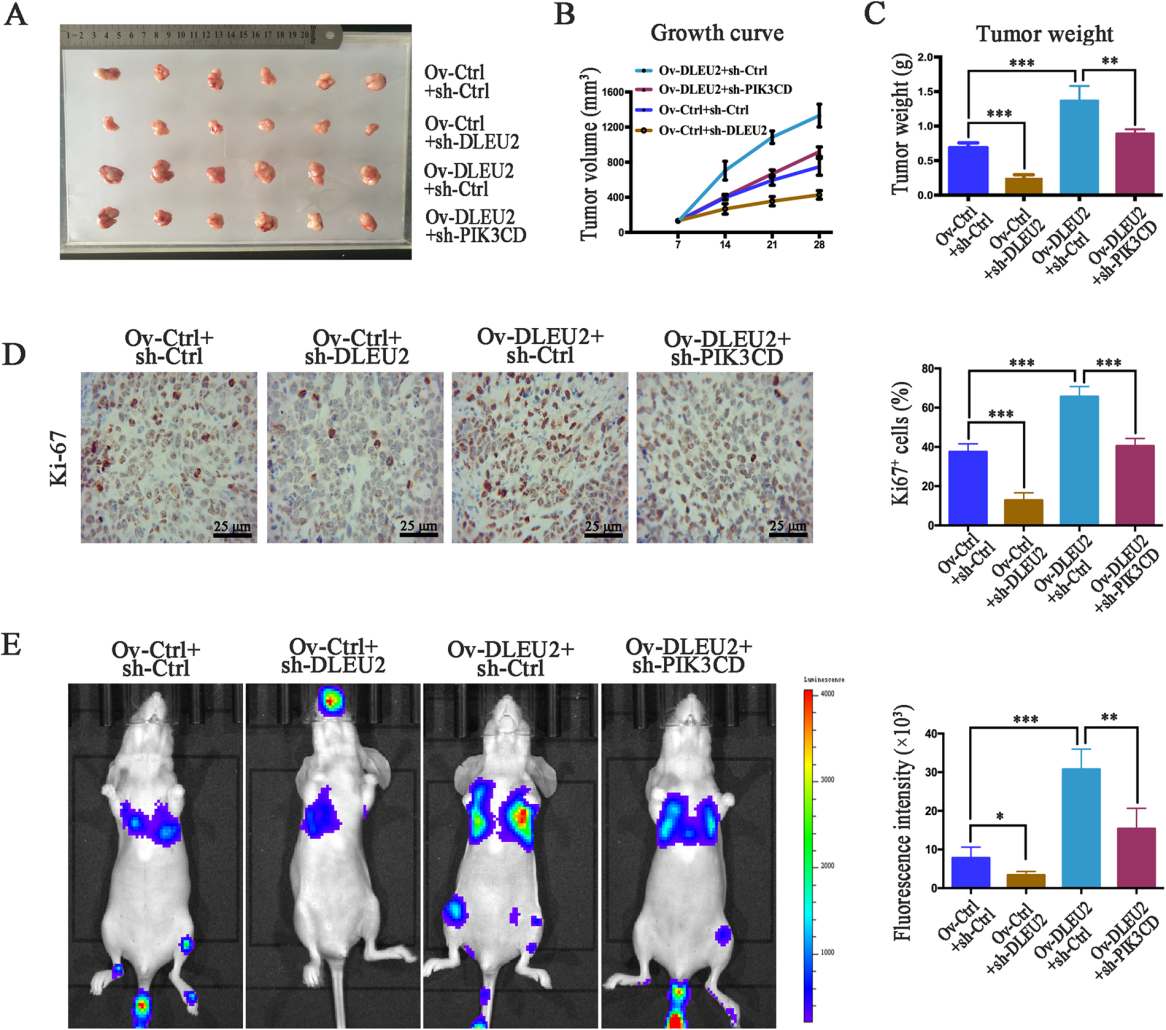
DLEU2 increases PIK3CD expression to promote LSCC cell proliferation and metastasis in vivo. **a** Tumor size was measured when the animal was sacrificed. **b**, **c** The growth curve (**b**) and tumor weights (**c**). **d** Proliferative ability was evaluated by immunohistochemistry of Ki-67 (original magnification: ×200; scale bar = 25 µm). **e** Left: representative bioluminescent images obtained at 4 weeks after tail vein injection of Hep2 cells. Right: quantification of bioluminescent imaging signal intensity, as detected using a noninvasive in vivo imaging system. *P < 0.05, **P < 0.01, ***P < 0.001.

To examine the effect of DLEU2 on LSCC growth and metastasis in vivo, nude mice were implanted with cotransfected Hep2 cells expressing different levels of DLEU2 and PIK3CD. Compared with the control group, implantation of cells overexpressing DLEU2 caused a significantly increased tumor growth (tumor volume and weight) and proliferation. However, DLEU2 knockdown remarkably suppressed tumor growth, as indicated by a decreased tumor volume and weight, and inhibited tumor proliferation (Fig. 7a–d). Moreover, compared with the tumors overexpressing DLEU2 only, tumors with both DLEU2 overexpression and PIK3CD knockdown showed suppressed tumor growth, decreased tumor volume and weight, and inhibited proliferation (Fig. 7a–d).

We next injected these engineered cells into a mouse model of metastatic lung cancer via tail vein injection. The fluorescence intensity of the lung tissue was measured 4 weeks after the injection. The fluorescence intensity was significantly lower in the DLEU2 knockdown group and higher in the DLEU2 overexpression group, compared with the control group (Fig. 7e), indicating that DLEU2 promoted LSCC metastasis. Compared to the animals overexpressing DLEU2 alone, mice with inhibition of PIK3CD had significantly decreased DLEU2-induced metastasis (Fig. 7d). These results demonstrate that DLEU2 promotes the growth and metastasis of LSCC cells through the upregulation of PIK3CD in vivo.

## Discussion

Emerging evidence has demonstrated that up to 90% of transcripts transcribe to produce noncoding RNAs (ncRNAs), including miRNAs and lncRNAs^6^. It is well accepted that lncRNAs regulate the expression of protein-coding genes via an accurate regulatory ceRNA network, where lncRNAs and miRNAs suppress each other^7^. Recently, multiple studies have indicated that the lncRNA−miRNA networks are vital to a variety of malignant processes in cancers^10,11^. Therefore, lncRNA dysregulation is thought to be responsible for various malignant processes, making these molecules attractive therapeutic targets^6,8,9,30^. For instance, our previous study demonstrated that the upregulated lncRNA PEG10 could promote the tumorigenic activities of proliferation, invasion and migration in hypopharyngeal squamous cell carcinomas^21^. However, only a few lncRNAs have been identified to play crucial roles in the tumorigenesis and development of LSCCs^14–17^. In the present study, we identified a set of lncRNAs that involved in the progression of HNSCCs, based on the topological features of the competitive network and the significant subpathways^18^. The top three subpathways that were competitively mediated by lncRNAs were 04012_1 ErbB signaling pathway, 04720_1 Long-term potentiation, and 05169_2 Epstein−Barr virus infection, respectively (Supplementary Fig. S1). Among these lncRNAs, we hypothesized that DLEU2 was the key lncRNA in HNSCCs.

To verify this hypothesis, we investigated the clinical significance and functional roles of DLEU2 in LSCCs. The results indicated that DLEU2, as an independent prognostic factor, was significantly upregulated in LSCC specimens and was correlated with the malignant progression of LSCCs (Fig. 1d–i). These results were consistent with the TCGA data (Fig. 1c) and suggested that DLEU2 could be a potential molecular biomarker to distinguish the malignancies of LSCCs. Functionally, DLEU2 modulated the malignant behaviors (including the proliferation, invasion, and migration) in vitro and promoted the growth as well as metastasis of the xenografted tumors in vivo (Figs. 2, 7), suggesting that DLEU2 could be a potential therapeutic target of LSCCs.

Aberrant expression of DLEU2 has been frequently identified in cancers and plays crucial roles in the modulation of multiple oncogenic properties^31–39^. For example, DLEU2 expression is inversely associated with survival among patients with pancreatic cancer, esophageal adenocarcinoma, and clear cell renal cell carcinoma^36–38^. In these studies, DLEU2 was shown to be essential in modulating the proliferation, invasion, and migration of tumor cells. These findings are consistent with those reported here and demonstrate that DLEU2 plays a crucial role in the tumorigenesis of multiple cancers. Intriguingly, Xie et al.^40^ have shown that the DLEU2 levels are significantly decreased in laryngeal carcinoma tissues. The upregulation of DLEU2 induces decreased cell proliferation, migration, and invasion, compared to the control samples. These findings seem to contradict our results. However, we repeatedly analyzed the TCGA data as well as our experimental results and confirmed that the expression of DLEU2 in LSCCs was higher than that in the paracarcinoma tissues (Fig. 1c, d).

Based on the lncRNA–mRNA interactions, PIK3CD, PPP3R1, and PPP1CC were identified as potential targets of DLEU2 (Supplementary Fig. S3). Therefore, we detected the expression signatures of PIK3CD, PPP3R1, and PPP1CC in LSCC specimens. However, the analysis of LSCC specimens did not reveal significant correlations of either PPP3R1 or PPP1CC with the prognosis or tumor malignancy (data not shown), while an increased PIK3CD expression was significantly correlated with tumor progression and the clinical outcomes (Fig. 3e–h, j). In addition, Spearman’s correlation analysis indicated that PIK3CD expression was positively correlated with the expression of DLEU2 in LSCC clinical specimens (Fig. 3d). Therefore, we used PIK3CD as a potential target of DLEU2 for subsequent analyses.

As a target gene of multiple tumor suppressor miRNAs, numerous reports have indicated that PIK3CD is upregulated and involved in tumorigenesis^24–29^. In glioblastomas and colorectal cancers, PIK3CD is reported to be upregulated and promote cell growth, migration, and invasion through the activation of Akt signaling^24,25^. Consistent with these studies, our study showed that Akt signaling was upregulated in cells overexpressing DLEU2 and that PIK3CD silencing attenuated the upregulation of Akt signaling. The involvement of DLEU2 in LSCC cell proliferation, invasion, and migration might be partially explained by activation of the Akt signaling targets. However, the effect of DLEU2 on the upregulation of Akt signaling could not be fully compromised by PIK3CD silencing, suggesting that other target genes might be involved in the activation of Akt signaling induced by DLEU2. Furthermore, a previous study has found that the upregulation of PIK3CD increases LSCC metastasis, which is also consistent with our findings^28^. We demonstrated that the inhibition of PIK3CD partially abolished the tumorigenic roles of DLEU2, both in vitro and in vivo (Figs. 6, 7). These data indicate that other target genes may participate in the modulation of the malignant properties induced by DLEU2 in LSCCs. Thus, the role of DLEU2 in LSCC malignancies needs further investigation.

As competitive endogenous RNAs sponge miRNAs, the cancer-related functions of DLEU2 are closely related to the transcription of its intronic miRNAs^41^. For instance, the lncRNA DLEU2 acts as a sponge for miR-186-5p and regulates the expression of PDK3 in the progression of human gliomas^42^. In addition, an elevated DLEU2 expression promotes tumor aggressiveness by regulating the miR-455/SMAD2 axis in pancreatic cancer^37^. Moreover, the downregulated target of DLEU2, miR-30a-5p, suppresses the epithelial–mesenchymal transition in clear cell renal cell carcinoma via the repression of ZEB2^38^. In view of the lncRNA–miRNA–mRNA interactions, we performed bioinformatics analysis and found that the miR-30 family (miR-30a-5p, miR-30b-5p, miR-30c-5p, miR-30d-5p, and miR-30e-5p) and the miR-374 family (miR-374a-5p and miR-374b-5p) were potential targets of DLEU2, which was consistent with a previous study^43^. According to the TargetScan database, the 5ʹ-UTR of PIK3CD harbors a putative miR-30c-5p binding site (Fig. 4e). Therefore, we speculated that DLEU2 increased PIK3CD expression by competitively sponging miR-30c-5p and enhancing the malignant properties of LSCCs. As expected, DLEU2 acts as a ceRNA by sponging miR-30c-5p and decreases PIK3CD expression in LSCCs (Fig. 4a–d). Consistent with our study, Zhou et al.^43^ have reported that DLEU2 serves as an oncogene via the miR-30c-5p/SOX9 axis to facilitate cell proliferation and invasion in non-small-cell lung cancer. Moreover, we found that PIK3CD was also a direct target of miR-30c-5p and inversely regulated by miR-30c-5p in LSCCs (Fig. 4e–i). All of the above evidence enhanced our comprehension of the molecular mechanism of the DLEU2/miR-30c-5p/PIK3CD interactions, which played a critical role in regulating the aggressiveness of LSCCs.

Taken together, we adopted a novel subpathway strategy to identify lncRNAs that competitively regulate LSCC malignancies based on the information obtained from the TCGA database. We revealed that DLEU2 was an independent prognostic factor that promoted LSCC growth and metastasis by inducing competitive binding to miR-30c-5p, resulting in the upregulation of PIK3CD as well as Akt signaling. Thus, the lncRNA-DLEU2/miR-30c-5p/PIK3CD/Akt axis may be used as a novel prognostic biomarker and therapeutic target for LSCCs. The expression signatures and clinical significance of DLEU2 in other human HNSCCs require further investigation.

## Supplementary information

Supplementary materials and methods

Supplementary Figure Legends

Supplementary Figure S1

Supplementary Figure S2

Supplementary Figure S3

Supplementary Table S1

Supplementary Table S2
