# Supplementary material for: Long noncoding RNA DLEU2 predicts a poor prognosis and enhances malignant properties in laryngeal squamous cell carcinoma through the miR-30c-5p/PIK3CD/Akt axis
Source: Cell Death Dis. 2020 Jun 18;11(6):472. doi: 10.1038/s41419-020-2581-2 (PMC7303144; doi:10.1038/s41419-020-2581-2)
Supplement: Supplementary file 1 — Supplementary materials and methods [file 41419_2020_2581_MOESM1_ESM.docx]

**Supplementary materials and methods**

**Expression data processing**

The lncRNA expression profiles were downloaded from the TCGA HNSCC RNA-seq database, which contained 44 normal samples and 500 HNSCC samples. RNA sequence reads were aligned to the human reference genome hg19 and Ensembl genome v.82 using STAR v.2.4.2a. RNA sequence was knocked out when they met the following criteria: a) intron<30 or >500000; b) Non-classical, unannotated points; c) Mismatch tolerance >10. RNA Abundance quantification of each sample was carried out by Cufflinks v.2.2.1, combined with offset and multilevel correction. To obtain the Fragments Per Kilobase Million, Cffnorm command and default parameters worked together. Then, an expression profile contained 36284 genes were gained.

**Building of the lncRNA-mRNA competitively regulated interactions**

The miRNA-mRNA interactions and lncRNA-miRNA intersections were primarily collected from starBase v2.0, TarBase, mirTarBase, mir2Disease and miRecords (V4.0). Based on the shared miRNAs, lncRNA-miRNA-mRNA interactions were constructed. Briefly, candidate lncRNA-mRNA interactions were determined by the hypergeometric test of shared miRNAs with false discovery rate (FDR) < 0.05 and Jaccard Coefficient ranked at top 20%. The intersection between RNA expression profiles and candidate lncRNA-mRNA interaction was considered as lncRNA-mRNA competitively regulated Interaction.

**Evaluation of lncRNA-mRNA intersections and embedding of lncRNAs to different pathways**

Based on the matched lncRNA and mRNA expression profiles, Spearman’s correlation coefficient was used to evaluate the lncRNA-mRNA intersections. After Fisher's z transformation, those *P* < 0.01 were considered to be significant lncRNA-mRNA intersections under specific conditions in our study.

The mRNAs of lncRNA-smRNA interactions enrichment analysis was carried out based on the KEGG pathway methods. Different pathways were identified by the Fisher’s test with the threshold of *P* < 0.01. In order to reconstruct the lncRNA competitively regulated signal pathways (LRSP), lncRNAs were embedded in the different pathways.

**Identification and evaluation of the LRSP subpathways**

We reconstructed subways graphs by embedding lncRNAs to different pathways. LncRNA and miRNA participating in the competing regulation were deemed important nodes, which could assist us with precisely locating subpathways. Important nodes were linked to LRSP and “lenient distance” similarity and network topology feature were used to identify the LRSP subways^16^. The detailed processes were described below. Firstly, the shortest path between any two important nodes of LRSP was calculated, if the count of molecules nodes between each pair of signatures was no more than k (k=1), then they will be merged into one node. Next, the number of nodes of the molecule sets in pathways were calculated, whose nodes count was no smaller than m (m=8) were considered as candidate LRSP subpathways.

In the present study, Wallenius approximation methods were used to estimate the significance of candidate subpathways^17^. According to the following formula (1), subpathway weight was calculated:
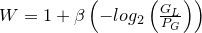
, where P_G_ represents the number of mRNAs of the specific subpathway, while, G_L_ was mRNA counts competitively regulated by lncRNAs within this subpathway. β is a parameter with β=1.

Consequently, adjusted p values were computed based on the formula (2):
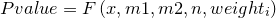
. Detailed meaning of parameters appeared above as follows: x, the number of mRNAs in lncRNA-mRNA intersections enriched in the given subpathway; n, the total mRNAs is also the background mRNA; m1, the number of background mRNA involved in the given subpathway; m2, the number of interesting background mRNAs annotated into lncRNA-mRNA intersections; and w was the weight of this pathway. The specific algorithm was referred to R package^17^.

**Identification of hub lncRNAs and key lncRNAs based on LRSP subpathway and network**

LncRNA-mRNA interaction networks were firstly constructed based on the LRSP subpathway evaluation. Topological features were further studied to find key nodes in the network. LncRNAs, whose degree was bigger than average degree values were considered as hub lncRNAs.

After evaluating the LRSP subpathway, the top three members were determined. The overlapping molecules contained in these pathways were selected and build a dataset. We took the intersection of this data set and hub lncRNAs. LncRNAs contained in this intersection were regarded as key lncRNAs.

**Human tissue specimens**

A total of 66 LSCC tissues and paired adjacent normal control tissues were collected from Shandong Provincial Hospital Affiliated to Shandong First Medical University between December 2011 and September 2013 for experimental verification of predicted key lncRNAs. All the LSCC tissues were confirmed by pathologic examination. All patients were staged according to the tumor node metastasis (TNM) classification of the International Union Against Cancer and followed up every three months after surgery until September 2018. No patients received chemotherapy or radiotherapy before surgery but all the patients received postoperative radiotherapy.

**Western blotting**

Total proteins were extracted using RIPA (Beyotime, Shanghai, China), separated by 8% sodium dodecyl sulphate-polyacrylamide gel electrophoresis (SDS-PAGE), and then transferred to polyvinylidene difluoride membranes (Millipore, Bedford, MA, USA). Followed by blocking with skimmed milk powder, the membranes were incubated overnight at 4 °C with the corresponding primary antibodies: PIK3CD (Santa Cruz Biotechnology, Dallas, TX, USA, 1:1000), AKT, phosphorylated (phospho)-AKT (Ser473) (p-Akt, Cell Signaling Technology, Beverly, MA, USA, 1:1000 and 1:500, respectively), CyclinD1 and Cyclin E1 (Abcam, Cambridge, MA, USA, CCND1, 1:5000 and CCNE1, 1:2000), MMP-2 and MMP-7 (R&D Systems, Minneapolis, MN, 1:1000 for both) and β-actin (Beyotime, Shanghai, China, 1:1000). After washing with TBST buffer, the immunostained protein bands were incubated with a Horseradish peroxidase (HRP)‐conjugated secondary antibody for 1 hour at room temperature. The resulting protein blots were washed with TBST buffer and visualized by ECL Kit (Pierce, Thermo Fisher Scientific, IL, USA). β-acting was used as internal reference.

**Immunohistochemical staining**

The primary antibodies for the immunohistochemical staining were used as follows: PIK3CD, p-AKT, CCND1, CCNE1, MMP-2, MMP-9 (all described above), and phospho-mTOR (Cell Signaling Technology, 1:500).

**The colony formation assay**

For colony formation assay, LSCC cells with different expression levels of DLEU2 or PIK3CD were seeded into 6-well plates and cultured for 10 days. Colonies were fixed with methanol, stained with 0.1% crystal violet and counted under an inverted microscope. The results were repeated three times.

**Migration and invasion assays**

Migration and invasion assays were performed at 48 h after transfection. In brief, 3 × 10^5^ cells were seeded into the upper chamber of the 24-well Millicell chambers (8-μm pore size, millepore) with Matrigel (BD Biosciences, CA, USA). The upper chamber was plated in serum-free media and the lower was added with 10% FBS media. After 24 hours of incubation, chambers were removed and cells were fixed in 4% paraformaldehyde, and then stained with 1% crystal violet. The migrated or invaded cells were imaged and counted using an inverted microscope. The results were repeated three times.

**Dual-luciferase reporter gene assay**

The putative miR-30c-5p binding sequences of the wild-type (WT) 3’UTR or mutant (MUT) 3’UTR of DLEU2 and PIK3CD were amplified and subcloned into pGL3 luciferase reporter vector (Promega, Madison, WI, USA), respectively. LSCC Cells were seeded in 96-well plates and cotransfected with either miR-30c-5p mimics or control-mimics and the WT/MUT 3′-UTR of DLEU2 or PIK3CD vector using the Lipofectamine 2000 reagent according to the manufacturer’s instructions. At 48 h after transfection, the cells were harvested and normalized to luciferase activity by the dual-luciferase reporter assay system (Promega). The experiments were repeated three times.

***In vivo* tumor metastatic model**

BALB/c nude mice (female, 4-5-week-old, 18-20g) were obtained from the Model Animal Research Center of Shandong University. Hep2 cells were cotransfected with lentivirus vectors of Ov-Ctrl + sh-Ctrl, Ov-Ctrl + sh-DLEU2，Ov-DLEU2 + sh-Ctrl and Ov-DLEU2 + sh-PIK3CD, respectively. All animals were randomly divided into the above four groups (n = 6 per group). For the *in vivo* lung metastasis assay, Hep2 cells (2 × 10^6^) infecting with the above vectors were labeled with firefly luciferase and injected into the tail vein of mice. The metastatic progression was monitored weekly and quantified by a noninvasive bioluminescence In-Vivo Imaging System (IVIS, Xenogen, Caliper Life Sciences, Hopkinton, MA, USA).
