# Supplementary material for: Long noncoding RNA DLEU2 predicts a poor prognosis and enhances malignant properties in laryngeal squamous cell carcinoma through the miR-30c-5p/PIK3CD/Akt axis
Source: Cell Death Dis. 2020 Jun 18;11(6):472. doi: 10.1038/s41419-020-2581-2 (PMC7303144; doi:10.1038/s41419-020-2581-2)
Supplement: Supplementary file 2 — Supplementary Figure Legends [file 41419_2020_2581_MOESM2_ESM.docx]

**Supplementary Figure Legends**

**Supplementary Fig. S1 Identification of HNSCC-related lncRNAs using TCGA and multiple RNA databases.** A-C. The top three LRSP subpathways identified: 04012_1, ErbB signaling pathway (A); 04720_1, Long-term potentiation (B); 05169_2, Epstein-Barr virus infection (C). Circular patterns and triangle patterns represent mRNAs and lncRNAs, respectively.

**Supplementary Fig. S2 LncRNA-mRNA interaction networks.** Circular patterns, triangular patterns, and square patterns represent mRNAs, lncRNAs, and pathway IDs, respectively. Green triangles represent hub lncRNAs. Blue triangles represent non-hub lncRNAs.

**Supplementary Fig. S3 The interaction networks of key lncRNA-mRNAs.** Circular and triangular patterns represent mRNAs and lncRNAs, respectively. Red triangles represent key lncRNAs.
