# Supplementary figures and images for: Long noncoding RNA DLEU2 predicts a poor prognosis and enhances malignant properties in laryngeal squamous cell carcinoma through the miR-30c-5p/PIK3CD/Akt axis
Source: Cell Death Dis. 2020 Jun 18;11(6):472. doi: 10.1038/s41419-020-2581-2 (PMC7303144; doi:10.1038/s41419-020-2581-2)

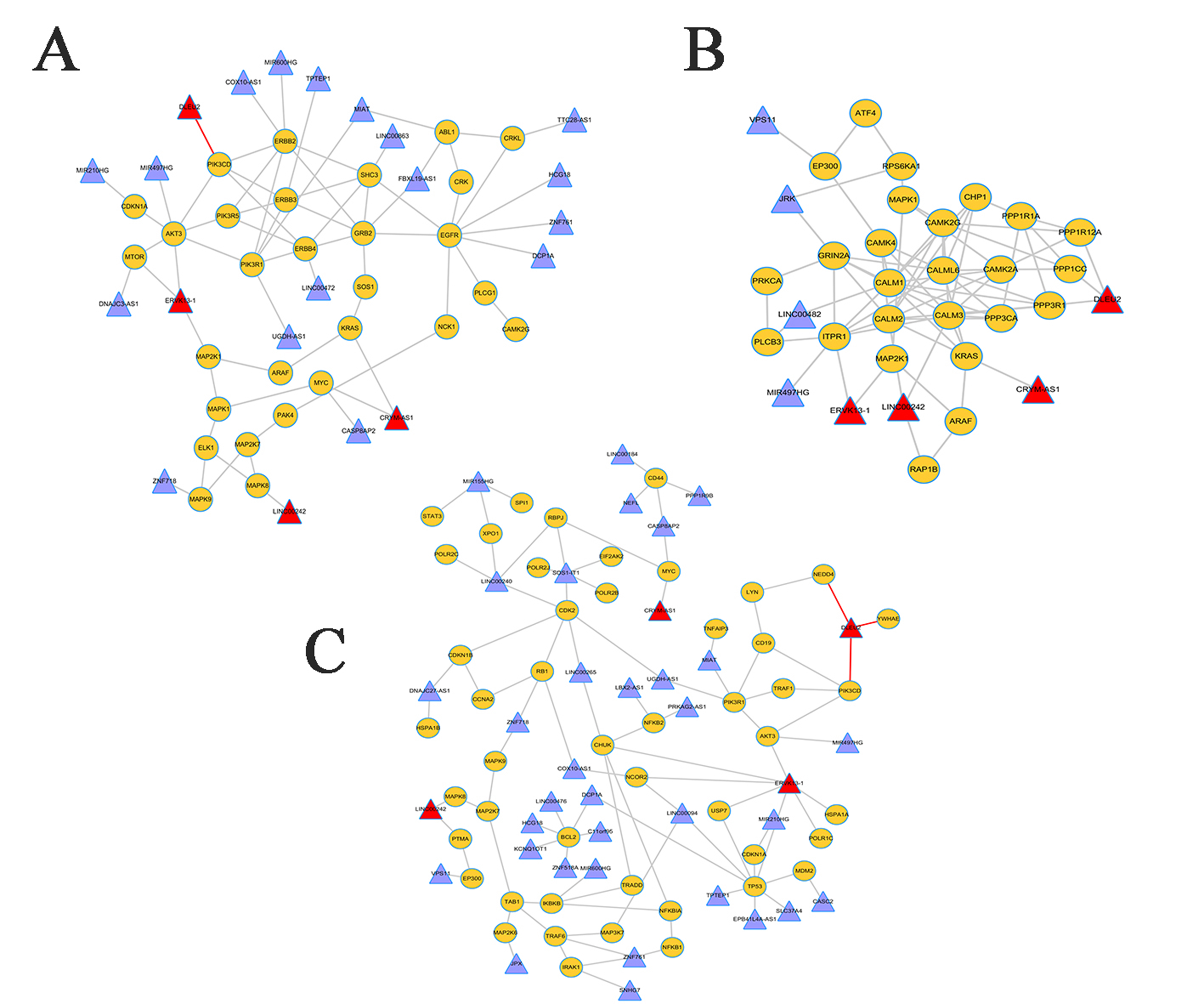

Supplement: Supplementary file 3 — Supplementary Figure S1 [file 41419_2020_2581_MOESM3_ESM.tif]

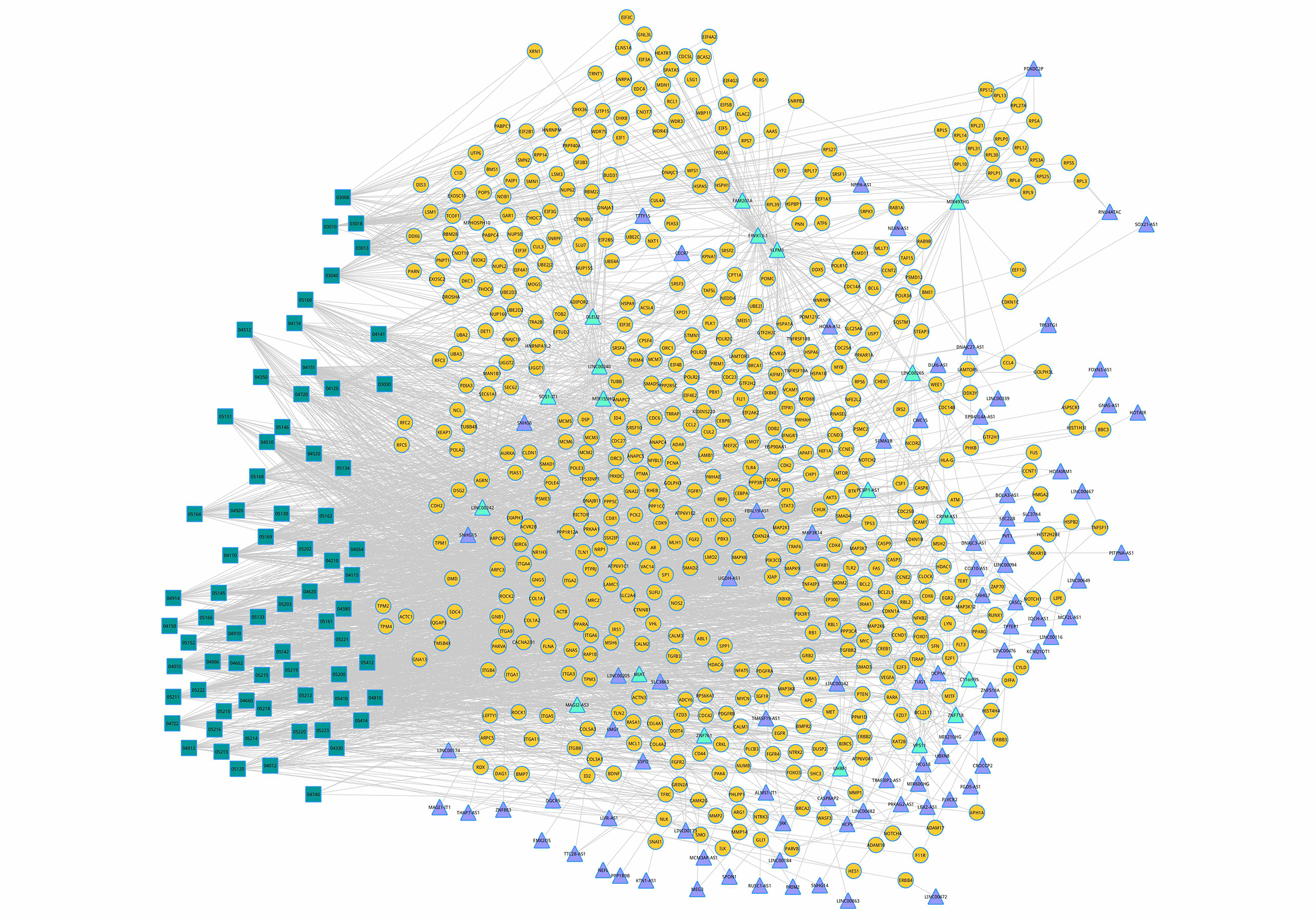

Supplement: Supplementary file 4 — Supplementary Figure S2 [file 41419_2020_2581_MOESM4_ESM.tif]

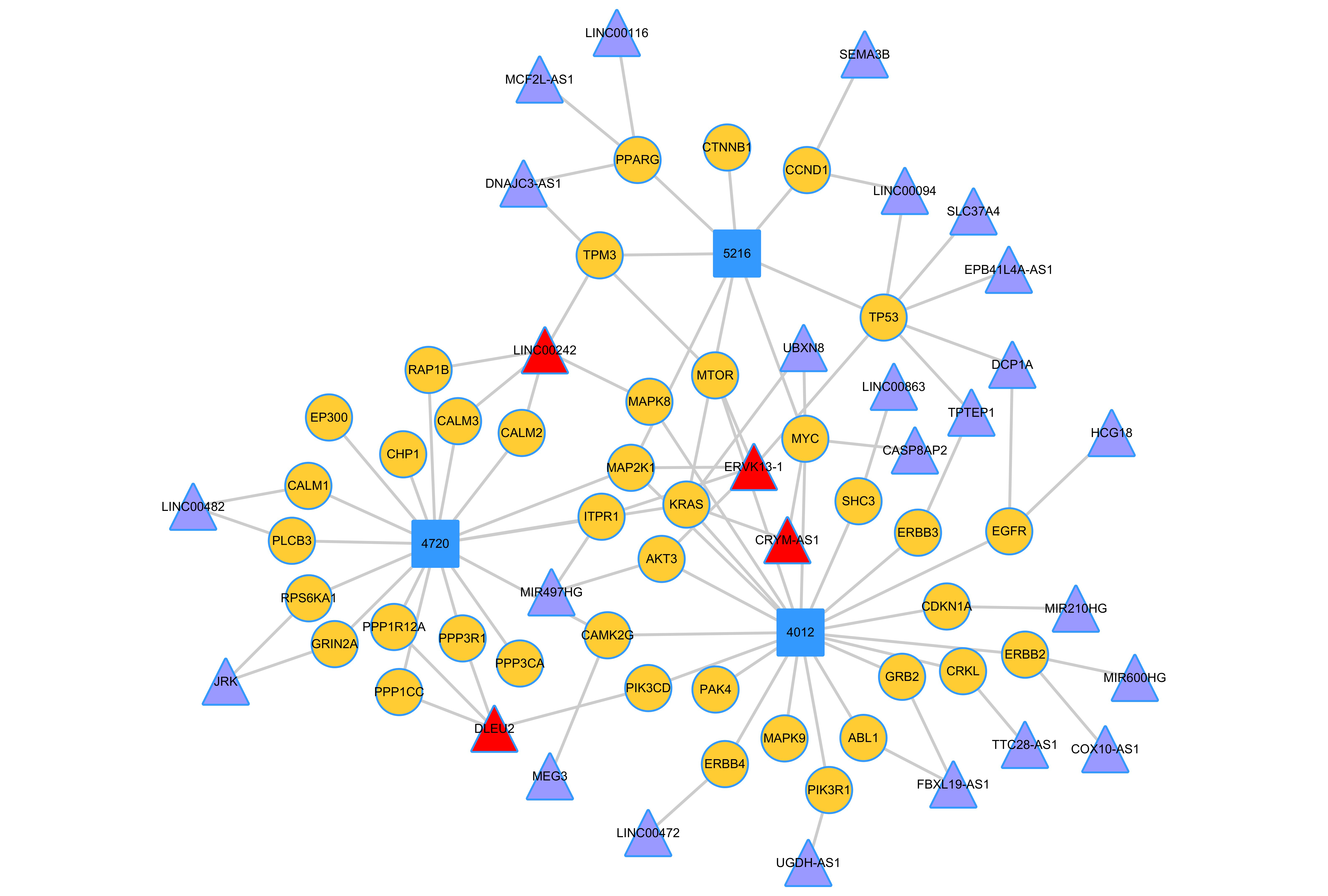

Supplement: Supplementary file 5 — Supplementary Figure S3 [file 41419_2020_2581_MOESM5_ESM.tif]
