# Supplementary material for: Long noncoding RNA DLEU2 predicts a poor prognosis and enhances malignant properties in laryngeal squamous cell carcinoma through the miR-30c-5p/PIK3CD/Akt axis
Source: Cell Death Dis. 2020 Jun 18;11(6):472. doi: 10.1038/s41419-020-2581-2 (PMC7303144; doi:10.1038/s41419-020-2581-2)
Supplement: Supplementary file 6 — Supplementary Table S1 [file 41419_2020_2581_MOESM6_ESM.docx]

**Supplementary Table S1 Primary primers for qRT-PCT**

| **Gene symbol** | **Primer sequence (5’-3’)** |
| --- | --- |
| **DLEU2** | F: TTAGTTGATGCCAAAGAGTTCC |
|  | R: ATCCATACTCCAAGTAACAGAC |
| **PIK3CD** | F: TCCAGAAACATCTAAGCTGGA |
|  | R: GGAGGATCACTTGAAGCCA |
| **PPP3R1** | F: TCTTGTGGTATGGAACTATGTG |
|  | R: ATCCGCATCAAAGTGTGAG |
| **PPP1CC** | F: TGTAAACATCCTACACTCTCAGCAA |
|  | R: GCTGTCAACGATACGCTACGTAACG |
| **miR-30c-5p** | F: TGTAAACATCCTACACTCTCAGCAA |
|  | R: GCTGTCAACGATACGCTACGTAACG |
| **CCND1** | F: GCTGCGAAGTGGAAACCATC |
|  | R: CCTCCTTCTGCACACATTTGAA |
| **CCNE1** | F: ATGAAGAAGTTGAACCATGCCA |
|  | R: CCTCCAGAACAGTATTCCATTGC |
| **MMP2** | F: CCACTGCCTTCGATACAC |
|  | R: GAGCCACTCTCTGGAATCTTAAA |
| **MMP7** | F: GAGATGCTCACTTCGATGAGG |
|  | R: CCTAGACTGCTACCATCCGTC |
| **β-actin** | F: CGAGAAGATGACCCAGATCATG |
|  | R: GTGAAGCTGTAGCCGCGCTCGG |

F: forward primer; R: reverse primer.
