# Supplementary material for: Long noncoding RNA DLEU2 predicts a poor prognosis and enhances malignant properties in laryngeal squamous cell carcinoma through the miR-30c-5p/PIK3CD/Akt axis
Source: Cell Death Dis. 2020 Jun 18;11(6):472. doi: 10.1038/s41419-020-2581-2 (PMC7303144; doi:10.1038/s41419-020-2581-2)
Supplement: Supplementary file 7 — Supplementary Table S2 [file 41419_2020_2581_MOESM7_ESM.docx]

**Supplementary Table S2 Subpathways based on lncRNA-mRNA expression profiling and pathway** **topology**

| **Pathway** | **Pathway**  **Name** | **Molecule Ratio**  **(m2/x)** | **Bg Ratio**  **(m1/n)** | ***P*-value** | **Weight** | **Fdr** |
| --- | --- | --- | --- | --- | --- | --- |
| **04012_1** | ErbB signaling pathway | 19/782 | 31/26232 | 0.00E+00 | 1.706269 | 0.00E+00 |
| **04720_1** | Long-term potentiation | 17/782 | 27/26232 | 0.00E+00 | 1.667425 | 0.00E+00 |
| **05169_2** | Epstein-Barr virus nfection | 46/782 | 68/26232 | 0.00E+00 | 1.661198 | 0.00E+00 |
